# Minimal access mediastinal surgery: One or two lung ventilation?

**DOI:** 10.4103/0972-9941.59308

**Published:** 2009

**Authors:** Karamollah Toolabi, Ali Aminian, Mihan J Javid, Rasoul Mirsharifi, Abbas Rabani

**Affiliations:** Department of Surgery, Imam Khomeini Hospital, Tehran University of Medical Sciences, Tehran, Iran; 1 Department of Anaesthesiology, Imam Khomeini Hospital, Tehran University of Medical Sciences, Tehran, Iran

**Keywords:** Double lumen tube, hyperhidrosis, myasthenia gravis, single lumen tube, thoracoscopy, video-assisted thoracoscopic surgery

## Abstract

**BACKGROUND:**

Minimal access mediastinal surgery (MAMS) is usually performed under general anaesthesia with double lumen tubes (DLT). The aim of this study is to evaluate two lung ventilation through single lumen tubes (SLT) during thoracoscopic sympathectomy for hyperhidrosis and thoracoscopic thymectomy for myasthenia gravis.

**METHODS:**

In this prospective non-randomized study, MAMS was performed in 58 patients with hyperhidrosis and 42 patients with myasthenia gravis, from January 2002 to December 2008. Patients were intubated with a DLT or SLT, 50 patients in each group. In the DLT group, endobronchial tubes were placed using the traditional blind approach and one lung ventilation was confirmed clinically. In the SLT group, the hemithorax was insufflated with CO2 in conjunction with two-lung anaesthesia. All the patients were evaluated for haemodynamic stability, oxygen saturation of haemoglobin (Spo2), end-tidal Pco2 (ETPco2), times required for intubation and surgery, satisfaction of surgeon with regard to exposure and postoperative complications.

**RESULTS:**

In the SLT group, all the patients had stable haemodynamic and ventilation parameters. In the DLT group, haemodynamic instability occurred in two, decrease in Spo2 in four and increase in ETPco2 in three patients. One patient in the DLT group developed vocal cord granuloma two months later. Time required for surgery and the surgeon's opinion with regard to exposure were similar for both groups.

**CONCLUSION:**

Thoracoscopic surgery when used in cases where a well-collapsed lung may not be essential, since surgery is not performed on the lung itself, does not require DLT. SLT is safe in MAMS. It provides good surgical exposure and decreases the cost, time and undesirable complications of DLT.

## INTRODUCTION

Hyperhidrosis is a dysregulation of the neural sympathetic control of the sweat glands, which leads to excessive and unpleasant sweating. The thoracic sympathetic chain is involved in the neural control of sweating in hyperhidrosis of the upper limbs. There are several ways to treat excessive sweating: antiperspirants, iontophoresis, anticholinergic medication, psychotherapy, botulinum toxin injections and surgery. Thoracoscopic sympathectomy (TS) offers a safe and durable option for patients with severe symptoms. It requires excision, electrocoagulation or application of surgical clips on the thoracic sympathetic ganglia between T_2_ and T_4_. In the past, this surgery required bilateral thoracotomies, and therefore, it was rarely indicated. However, TS is a minimally invasive technique, which has created a lot of interest in recent years.[1]

Myasthenia gravis is an autoimmune disease characterized by muscle weakness and fatigue. Thymectomy is a radical, but effective treatment for this disease. The strongest evidence of its efficacy is a computer-matched series, which has shown a reduction in myasthenia-related mortality from 44 to 14% and an increase in remission rate from 8 to 35%.[2] In recent years, thoracoscopic thymectomy (TT) has been described as being able to achieve the same functional improvement as median sternotomy or the transcervical approach, but with less morbidity.[3]

Video-assisted thoracoscopic surgery (VATS) is usually performed under general anaesthesia with one lung ventilation (OLV). Lung separators in the airway such as double lumen tubes (DLT) or bronchial blockers are essential tools.[4,5] An anatomical shunt as a result of the continued perfusion of a non-ventilated lung is the principal intraoperative concern during OLV.[6] The undesirable complications of endobronchial intubation are a decrease in oxygen saturation, increase in airway pressure, poor lung ventilation, airway injury and displacement of the tube during the operation.[7]

In this clinical trial, we evaluated the efficacy and safety of two lung ventilation through a single lumen tube (SLT) in minimal access mediastinal surgery (MAMS) including thoracoscopic sympathectomy (TS) for hyperhidrosis and thoracoscopic thymectomy (TT), for myasthenia gravis.

## MATERIALS AND METHODS

Fifty-eight patients with hyperhidrosis and 42 patients with myasthenia gravis were enrolled in this prospective nonrandomized study between January 2002 and December 2008. All the patients signed a written informed consent. A single surgical team performed all the operations, in cooperation with an experienced anaesthesiologist, intensivist and neurologist, for handling the perioperative care. After induction of anaesthesia, the patients were intubated with a DLT or SLT; there were 50 patients in each group. In the DLT group, left-sided endobronchial tubes were placed using the traditional blind approach and OLV was confirmed clinically. In the SLT group, the right hemithorax was insufflated with CO_2_ (up to a maximum pressure of 8 mmHg) in conjunction with two-lung anaesthesia. The patients were monitored with an electrocardiogram (ECG), non-invasive blood pressure, pulse oximetry and capnography during the operation.

We have described surgical techniques in detail previously.[8,9] In TS, patients are positioned supine in a semi-sitting position. The operation is usually performed with an insertion of three 5 mm ports. The sympathetic chain is visualized behind the parietal pleura. After identification of the stellate ganglion, the sympathetic chain is cauterized and divided from T_2_ to T_3_ for patients with predominantly palmar hyperhidrosis and from T_2_ to T_4_ for patients with predominantly axillary hyperhidrosis. Contralateral sympathectomy is performed in a similar manner, if necessary.[8,10]

In TT, The patient was turned 45° to the left. Operations were performed through the right-sided approach, with four thoracoscopic ports (two 10 mm and two 5 mm). A clear view of the entire mediastinum allowed a complete en bloc removal of all mediastinal fat and the thymus gland with its cervical horns. The dissection was begun at the inferior portion of the thymic gland just anterior to the phrenic nerve. By a combination of blunt and sharp dissection, all anterior mediastinal tissue was teased off the pericardium. Next, the gland was dissected off the retrosternal area and contralateral pleura with blunt dissection. After visualization of the innominate vein, the thymic vein(s) were double-clipped. Then, dissection was carried cephalad to the innominate vein until the superior horns of the thymus were identified. One chest tube was left in place, in one port, and the remaining incisions were closed.[9]

At the end of the surgery, the lungs were hyperinflated to reverse atelectasis. The patients were routinely sent to the intensive care unit. Blood pressure < 30% of baseline, heart rate > 120 beats / minute, oxygen saturation of haemoglobin (Spo_2_) < 90%, end-tidal Pco_2_ (ETPco_2_) > 45 mmHg during the operation, times required for intubation and surgery, and perioperative complications were recorded. At the end of the operation, the surgeon was asked to score from 1 to 5 on the appropriateness, surgical view and working field during the operation. Statistical analysis was performed with a t-test. A *P* value of < 0.05 was considered statistically significant.

## RESULTS

All the procedures were performed successfully, with no conversion to open surgeries.

All patients in the SLT group had straightforward intubation and operation. There was no unsuccessful or second try intubation in the SLT group. No desaturation, Pco_2_ rising, haemodynamic disturbances, or significant complications occurred [Table 1].

**Table 1:** Comparison of SLT and DLT in MAMS (TS and TT)

	DLT group	SLT group
N (TS / TT)	50 (29 / 21)	50 (29 / 21)
Sex (M / F)	15 / 35	17 / 34
Age (years) (Mean)*	26.7	23.8
Second-try intubation	4	0
Poor lung isolation	2	-
Haemodynamic instability**	2	0
Spo_2_ < 90%	4	0
ETPco_2_ > 45 mmHg	3	0
Time required for intubation (min) (Median)	11	2
Duration of surgery (TT) (min) (Mean ± SD)*	164 ± 33	171 ± 29
Duration of surgery (TS) (min) (Mean ± SD)*	38 ± 12	40 ± 11
Surgeon opinion about exposure (Mean ± SD)*	3.8 ± 0.5	3.5 ± 1.1
Significant complications related to intubation		
Atelectasis	2	1
Vocal cord granuloma	1	0

* *P* > 0.05

** Define as blood pressure < 30% of baseline or heart rate > 120 beats / min, MAMS: minimal access mediastinal surgery; DLT: double lumen tubes; SLT: single lumen tubes; TS: thoracoscopic sympathectomy; TT: thoracoscopic thymectomy; Spo_2_: oxygen saturation of haemoglobin; ETPco_2_: end-tidal Pco_2_

In the DLT group, the first try for intubation failed in four patients. Poor lung isolation was observed in two patients. Haemodynamic instability occurred in two, decrease in Spo_2_ in four and increase in ETPco_2_ in three patients [Table 1].

The median time for placement and securing of an SLT averaged two minutes, compared to 11 minutes for a DLT. Time required for surgery was the same for both groups [Table 1].

Surgeon opinion about exposure during operation was not significantly different between the two groups (3.8 ± 0.5 for the DLT group and 3.5 ± 1.1 for the SLT group, *P* value = 0.2).

There was no perioperative mortality. Overall, 11 significant complications occurred. Nine of these complications occurred in TT. Two patients had atelectasis, one had aspiration pneumonia and four had postoperative myasthenic crisis, which required prolonged intubation and assisted ventilation. Two patients developed postoperative hoarseness. One patient in the DLT group had granuloma of the vocal cord, which was excised two months later. The other had intraoperative injury to the right recurrent laryngeal nerve. The other significant morbidity was temporary right upper extremity weakness subsequent to brachial plexus injury because of poor intraoperative positioning. During TS, one patient developed significant atelectasis and one had bleeding from the intercostals vessels.

## DISCUSSION

Although thoracoscopy was initially performed for diagnostic purposes, it later evolved into therapeutic procedures. The benefits of VATS include less postoperative pain, less pulmonary trauma, shorter hospitalizations, better cosmetic results and improved patient satisfaction. Both TT for myasthenia gravis and TS for hyperhidrosis have been proved as effective treatments.[1,3,8,9]

Several techniques have been suggested for lung ventilation during VATS, including OLV with DLT or bronchial blockers, jet ventilation, periods of apneic oxygenation, and two lung ventilations with SLT.[10,11] Various factors need to be taken into account to decide which method is most appropriate. Most of anaesthesiologists prefer OLV to provide the appropriate working field and facilitate the surgical process by elimination of lung movement. However, a DLT has complications and cost.

Physiological changes during VATS largely result from OLV and patient positioning. OLV decreases the surface area available for gas exchange and results in loss of normal pulmonary autoregulation. Stopping ventilation to a lung increases the proportion of the cardiac output that is not oxygenated (shunt fraction). Normally the shunt is in the region of 20 – 28%, irrespective of whether the anaesthetic is volatile agent- or intravenous agent-based. Increasing the inspired oxygen realistically does not have any effect above this value. Even though it usually reflects a ventilation problem, surgery should not continue until oxygen saturations are consistently above 90%.[6] OLV also increases pulmonary vascular resistance and right heart work. Lateral decubitus positioning minimizes the effects of OLV, in which a gravity-induced decrease in blood flow through a non-ventilated lung improves the ventilation-perfusion relationship. Ultimately, the tolerance of OLV is dependent on the preparation of the patient for surgery, the patient's pulmonary reserve and comorbidities.[10–12]

During OLV, as mentioned earlier, intrapulmonary shunting may result in changes in systemic oxygenation. These modest decreases in systemic oxygenation are accepted with the assumption that tissue oxygenation remains adequate. However, there are limited data examining the end-organ effects of the alterations in respiratory and cardiovascular function that may occur during OLV. Tobias *et al,* performed an observational study in order to monitor the brain tissue oxygenation with near infrared spectroscopy during OLV for thoracic procedures. They noted that changes in brain tissue oxygenation frequently occurred without changes in haemodynamic or ventilatory parameters. The incidence of significant cerebral desaturation was higher in this group of patients than demonstrated in other studies. They concluded that the use of OLV may place patients at a higher risk for cerebral desaturation.[13]

Several undesirable complications of endobronchial intubation with DLT have been reported in literature.[7,14–19] In one large series the rate of some complications of endobronchial intubation are decreased Spo_2_ 9%, increased airway pressure 9%, poor lung isolation 7%, air trapping 2% and airway trauma 0.4%.[7]

Tajima *et al,* reported the anaesthetic management for TT of 40 patients with myasthenia gravis. Operations were performed under general anaesthesia using DLT. Seven patients developed hypoxemia under OLV and needed bilateral lung ventilation or addition of continuous positive airway pressure (CPAP) to the non-dependent lung.[14]

Malpositioned DLTs have been reported to be found with bronchoscopy in 40 – 75% of the cases, despite auscultatory findings suggestive of correct placement.[15,16]

There are various reports that tracheobronchial injury is far more with the use of DLT than SLT. These lesions are usually located in the membranous portion of the trachea near the carina.[17,18] Although Lee *et al,* reported no statistical difference in the incidence of sore throat and hoarseness between DLT and SLT,[19] Knoll *et al,* showed higher cumulative number of days with hoarseness and sore throat, with use of DLT.[17]

Video-assisted thoracoscopic surgery when used in cases where a well-collapsed lung may not be essential, since surgery is not performed on the lung itself, does not require DLT. Satisfactory uses of SLT in thoracoscopic pleural biopsy[20] and mediastinal operations such as TS[21,22] and TT[23] have been shown. Of late we have reported a small randomized clinical trial, with 15 patients in each group, comparing SLT and DLT during TT. We found a successful application of SLT during TT for myasthenia gravis.[23] In this series with 100 patients who underwent MAMS, we did not observe any significant complications in the SLT group. Patients had stable haemodynamic and ventilation parameters. In contrast, haemodynamic disturbances, decreased Spo_2_, increased ETPco_2,_ failed first-try intubation and poor lung isolation were observed several times during OLV. Additionally, surgeon satisfaction with surgical exposure was similar for both the groups.

The main limitation of this non-randomized study was that the report was generated from a single team. Another problem with this study was the lack of use of fiberoptic bronchoscopy to confirm proper placement of the DLTs, which could lead to malpositioning and the subsequent consequences.

In conclusion, OLV with DLT is not necessary for MAMS. Use of SLT is a safe method of intubation for MAMS and provides good surgical exposure. It avoids the risk, time and cost of DLTs.
